# Maternal and Neonatal Outcomes After Assisted Reproductive Technology: A Retrospective Cohort Study in China

**DOI:** 10.3389/fmed.2022.837762

**Published:** 2022-04-05

**Authors:** Wen Tai, Lingmin Hu, Juan Wen

**Affiliations:** ^1^Department of Obstetrics, Nanjing Maternity and Child Health Care Hospital, Women’s Hospital of Nanjing Medical University, Nanjing, China; ^2^Department of Reproduction, Changzhou Maternal and Child Health Care Hospital, Changzhou Medical Center, Nanjing Medical University, Changzhou, China; ^3^Nanjing Maternity and Child Health Care Institute, Nanjing Maternity and Child Health Care Hospital, Women’s Hospital of Nanjing Medical University, Nanjing, China

**Keywords:** maternal outcomes, neonatal outcomes, art, cohort, China

## Abstract

**Background:**

With the progress of assisted reproductive technology (ART) and the increasing number of ART pregnancy, its safety has become the focus of attention. The present study aimed to explore the associations of ART pregnancy with maternal and neonatal outcomes, as compared with naturally pregnancy.

**Methods:**

This retrospective cohort study included all pregnant women who delivered at Women’s Hospital of Nanjing Medical University in 2011–2020. We compared maternal characteristics and pregnancy outcomes between group of ART pregnancy and group of naturally pregnancy using Logistic regression adjusted for confounders.

**Results:**

A total of 13,604 ART pregnancies and 198,002 naturally pregnancies were included. The proportion of ART pregnancies has increased every year for the past 10 years, peaking in 2020 (9.0%). Multivariable logistic regression analysis showed that the risks of gestational diabetes, preeclampsia, moderate or severe anemia, liver-related diseases, thyroid-related diseases, preterm birth, placenta previa, postpartum hemorrhage, and cesarean section were significantly increased in ART pregnancy. For neonatal outcomes, women conceived by ART were more likely to have twins or multiples, and the risk of stillbirth or abnormal development was also significantly increased. When restriction to singletons, these risks were reduced. And the effects of ART on the risk of premature rupture of membrane, cord entanglement, intrapartum fever, cesarean section, and stillbirth or abnormal development were more pronounced in singletons pregnancies compared with that in pregnancies of twins or multiples.

**Conclusion:**

Women conceived by ART were at increased risks of several adverse pregnancy outcomes compared with women conceived naturally. Multiple pregnancies could partly explain this phenomenon. For ART pregnancy, prenatal and intrapartum monitoring should be strengthened, and neonatal outcomes should be closely observed.

## Introduction

Since the birth of the first test-tube baby in 1978, assisted reproductive technology (ART) has become an effective treatment for infertility (1). With the progress of technology and provision of services, an increasing number of infants are born following ART therapy (1, 2). In developed countries, ART pregnancies account for 1.5–5.9% of all births (3–7), while in China, ART pregnancies account for 1.7% and are increasing year by year (2). ART is sometimes defined differently and is usually defined as the application of laboratory or clinical techniques to gametes and/or embryos for reproductive purposes, however it has been broadened to include not only *in vitro* procedures but also ovarian stimulation with gonadotropins or ovariotropic drugs (8, 9). As these and other reproductive technologies expand, leading to a substantial number of successful pregnancies and births, it is critical for prospective parents to understand the maternal and neonatal outcomes associated with ART.

Several studies have shown that ART pregnancies have an increased risk of multiple pregnancy and adverse pregnancy outcomes, including gestational diabetes, gestational hypertension, placenta previa, preterm birth, operative delivery, low birth weight, birth defects and perinatal mortality (10–16). However, other studies have concluded ART pregnancies do not have increased risks of adverse perinatal outcomes (7, 17–19). The incidences of small for gestational age, preterm birth and cesarean section are similar between ART and naturally pregnancies (13, 20). Nevertheless, pregnancy outcomes in ART pregnancies appear to be generally poorer due to the increased risk of multiple pregnancies. Multiple pregnancy is a post-processing confounding factor, which appears after ART treatment and may confound causal effects. Many previous studies did not adjust for maternal age, BMI and other confounding factors (12, 21–23). It is not clear whether the increased risk of adverse pregnancy outcomes is due to ART itself, multiple births, or potential infertility. At present, opinions are too far apart to reach a consensus.

The present retrospective cohort study was conducted to compare maternal and neonatal outcomes between ART and naturally pregnancies, and in addition to explore the association of ART with adverse pregnancy outcomes by stratifying on birth plurality and maternal age.

## Materials and Methods

### Study Design and Population

This retrospective cohort study included all pregnant women who delivered at Women’s Hospital of Nanjing Medical University in 2011–2020. The Women’s Hospital of Nanjing Medical University is the largest maternity hospital in Jiangsu province, China and delivers approximately 20,000 babies annually. After excluding women who had early abortions (≤12 weeks), or women who were discharged from care during pregnancy, a total of 211,606 pregnancies were included in the data analysis. Two cohorts were created: women who conceived by either intracytoplasmic sperm injection (ICSI), *in vitro* fertilization (IVF), ovulation induction (OI), gamete intra-fallopian transfer (GIFT), or artificial insemination (AI), were defined as group of ART pregnancy, and women who conceived naturally without ART, were considered as group of naturally pregnancy.

### Data Collection

We obtained all maternal and neonatal information from Hospital Information System (HIS) Database. Data were collected from standardized clinical forms and hospital records after maternity discharge to form the research database. All data were extracted and cleaned by using Natural Language Processing (NLP) technique (24). Maternal characteristics of all pregnancies were firstly extracted, including maternal age (year), height (cm), intrapartum weight (kg), parity, birthplace (Jiangsu province in China, other provinces in China or other countries), menstrual cycle (21–35 days, 36 days- or irregularity), abnormal pregnancy history and history of uterine fibroids. Maternal age was divided into five groups: <25, 25–29, 30–34, 35–39, ≥40 years. Intrapartum body mass index (BMI, kg/m^2^) was calculated as maternal intrapartum weight divided by the square of height, and classified into four groups: <25, 25–29.9, 30–34.9, ≥35 kg/m^2^. Parity did not include this pregnancy and was divided into 0 (nulliparae) and ≥1 (multiparae). Abnormal pregnancy history refers to a history of early abortion (≥2 times), intermediate and late abortion, abnormal development, or ectopic pregnancy.

We also used the HIS Database to obtain data on pregnancy complications, perinatal complications and neonatal outcomes. Data on pregnancy complications included gestational diabetes (fasting glucose concentrations ≥ 5.5 mmol/l or 2-h plasma glucose concentrations ≥ 8.0 mmol/l), preeclampsia (hypertension from 20 weeks’ gestation and proteinuria; severe preeclampsia was defined as preeclampsia with either a diastolic blood pressure ≥ 110 mmHg or proteinuria ≥ 5 g/day or both), anemia (hemoglobin < 100 g/l and hematocrit < 0.30; moderate or severe anemia was defined as hemoglobin < 90 g/l or 60 g/l), liver-related diseases (cholestasis, hepatitis, liver function damage, etc.) and thyroid-related diseases (hyperthyroidism, hypothyroidism, thyroiditis, etc.). Data on perinatal complications included hospitalization time (day), preterm birth (<37 weeks’ gestation), premature rupture of membrane, amniotic fluid pollution (clear as 0°, I°, II°, or III°), polyhydramnios (>2,000 ml in the third trimester), oligohydramnios (<300 ml in the third trimester), cord entanglement, torsion of cord, intrapartum fever (intrapartum temperature > 38°C), placenta previa, antepartum hemorrhage, postpartum hemorrhage (measured blood loss ≥ 500 ml) and delivery mode (spontaneous labor or cesarean section). And data on neonatal outcomes included gestational weeks in birth, offspring gender, birth weight (g), macrosomia (birth weight ≥ 4,000 g), twins or multiples, fetal distress, stillbirth or abnormal development (fetal malformation).

### Statistical Analyses

We compared maternal characteristics and pregnancy outcomes between group of ART pregnancy and group of naturally pregnancy. Continuous variables were described as mean and standard deviation ($\bar{x}$ ± *s*), and categorical variables were displayed as frequency (percentage). All comparisons between groups were conducted using standardized differences, which are not influenced by sample size and have been frequently used in previous large cohort studies (25–27). A standardized difference ≥ 0.1 indicates meaningful difference between groups. The association between ART using and pregnancy outcomes were evaluated by logistic regression analysis. The crude and adjusted odds ratio (OR) with 95% confidence intervals (95%CI) for pregnancy outcomes were calculated. Adjusted values were adjusted for maternal age, intrapartum BMI, parity, birth plurality and abnormal pregnancy history. All statistical analyses were two-sided and performed using R software (version 3.2.2).

## Results

**FIGURE 1:**
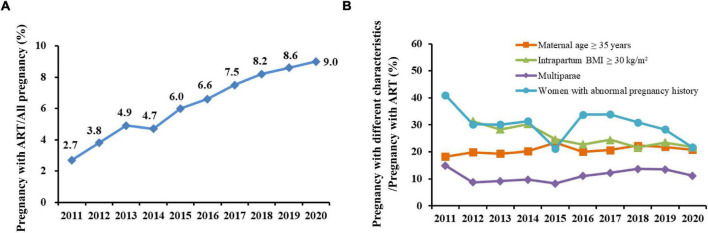
Yearly trends in percentage of assisted reproductive technology (ART) pregnancy and pregnancy with different characteristics from 2011 to 2020. **(A)** Percentage of ART pregnancy among all pregnancy; **(B)** Percentage of different characteristics among ART pregnancy.

A total of 211,606 women were included in this retrospective analysis, of whom 13,604 women conceived by ART as group of ART pregnancy, and 198,002 women conceived naturally without ART as group of naturally pregnancy. Over the past 10 years, the proportion of ART pregnancy has increased each year, reaching a peak in 2020 (9.0%) (Figure 1A). Of the ART pregnancy, the proportion of pregnant women over 35 years old and multiparae increased mildly, while the proportion of women with intrapartum BMI greater than 30 kg/m^2^ decreased slightly. And with the implementation of the universal two-child policy in 2015, the proportion of women with abnormal pregnancy history in ART pregnancy decreased sharply, and then increased rapidly (Figure 1B).

Maternal characteristics between ART and naturally pregnancy is summarized in Table 1. The mean maternal age and intrapartum BMI of women conceived by ART were significantly higher than those of women conceived naturally (standardized difference = 0.547 and 0.309, respectively). And women conceived by ART were more likely to be nulliparae (88.5% *vs.* 75.4%, standardized difference = 0.345), more likely to have a long or irregular menstrual cycle (15.2% *vs.* 7.9%, standardized difference = 0.230) and an abnormal pregnancy history (including early abortion, intermediate and late abortion, abnormal development, or ectopic pregnancy, 29.1% *vs.* 9.9%, standardized difference = 0.500), and more likely to have uterine fibroids (8.3% *vs.* 5.1%, standardized difference = 0.128). There were no significant standardized differences in maternal height and birthplace between the two groups.

**TABLE 1:** Maternal characteristics between naturally pregnancy and assisted reproductive technology (ART) pregnancy.

Maternal characteristics	All pregnancy (*n* = 211606)	Naturally pregnancy (*n* = 198002)	ART pregnancy (*n* = 13604)	Standardized difference
Maternal age [year, n (%)]	29.6 ± 3.9	29.5 ± 3.9	31.6 ± 3.9	0.547
<25	13274 (6.3)	13015 (6.6)	259 (1.9)	0.562
25-	104626 (49.4)	100616 (50.8)	4010 (29.5)	
30-	69047 (32.6)	62579 (31.6)	6468 (47.5)	
35-	21107 (10.0)	18711 (9.4)	2396 (17.6)	
40-	3550 (1.7)	3080 (1.6)	470 (3.5)	
Height (cm)	162.0 ± 4.7	162.1 ± 4.7	161.8 ± 4.7	0.066
Intrapartum weight (kg)	70.6 ± 9.1	70.5 ± 9.0	72.8 ± 9.9	0.252
Intrapartum BMI [kg/m^2^, n (%)]	26.9 ± 3.2	26.8 ± 3.1	27.8 ± 3.5	0.309
<25	43730 (28.9)	41489 (29.5)	2241 (20.7)	0.287
25-	84527 (55.8)	78580 (55.9)	5947 (55)	
30-	20845 (13.8)	18569 (13.2)	2276 (21.1)	
35-	2323 (1.5)	1977 (1.4)	346 (3.2)	
Parity [n (%)]				0.345
Nulliparae	161328 (76.2)	149292 (75.4)	12036 (88.5)	
Multiparae	50278 (23.8)	48710 (24.6)	1568 (11.5)	
Birthplace [n (%)]				0.039
Jiangsu province	196035 (93.9)	183445 (93.9)	12590 (93.0)	
Other provinces	12773 (6.1)	11823 (6.1)	950 (7.0)	
Menstrual cycle [day, n (%)]				0.230
21-	181015 (91.6)	169954 (92.1)	11061 (84.8)	
36- or Irregularity	16496 (8.4)	14521 (7.9)	1975 (15.2)	
Abnormal pregnancy history [n (%)]	22559 (11.2)	18681 (9.9)	3878 (29.1)	0.500
Early abortion (≥2 times)	17181 (8.5)	14496 (7.7)	2685 (20.2)	0.366
Intermediate and late abortion or abnormal development	4205 (2.1)	3459 (1.8)	746 (5.6)	0.200
Ectopic pregnancy	3234 (1.6)	2114 (1.1)	1120 (8.4)	0.347
With uterine fibroids [n (%)]	11295 (5.3)	10160 (5.1)	1135 (8.3)	0.128

*ART, assisted reproductive technology; BMI, body mass index.*

The incidences of pregnancy and perinatal complications in ART and naturally pregnancy was exhibited in Tables 2, 3. Statistically significant increases were noted in gestational diabetes (28.0%), preeclampsia (5.4%), thyroid-related diseases (13.7%), preterm birth (20.4%), placenta previa (8.5%), postpartum hemorrhage (19.2%) and cesarean section (75.5%) in ART pregnancy, compared to naturally pregnancy (standardized difference > 0.1). The occurring rates of anemia (25.9%), liver-related diseases (5.3%), polyhydramnios (3.4%), oligohydramnios (7.1%) and torsion of cord (3.3%) were also elevated in ART pregnancy, but with no significant difference (standardized difference < 0.1). In contrast, there was a decline in the incidences of premature rupture of membrane (21.7%) and amniotic fluid pollution (I°: 4.2%, II°: 3.5%, III°: 3.6%) in ART pregnancy (standardized difference > 0.1). We also analyzed neonatal outcomes between naturally pregnancy and ART pregnancy (Table 4). The mean birth weight of ART pregnancy was significantly lower than that of naturally pregnancy (standardized difference = 0.343). Moreover, significant rises of incidence were observed in twins or multiples (20.7%) and stillbirth or abnormal development (3.3%) in ART pregnancy (standardized difference > 0.1). No significant difference was noted in macrosomia and fetal distress between the two groups.

**TABLE 2:** The prevalence of pregnancy complications between naturally pregnancy and ART pregnancy.

Pregnancy complications [n (%)]	All pregnancy (*n* = 211606)	Naturally pregnancy (*n* = 198002)	ART pregnancy (*n* = 13604)	Standardized difference
Gestational diabetes	39498 (18.7)	35687 (18.0)	3811 (28.0)	0.239
Preeclampsia	5122 (2.4)	4382 (2.2)	740 (5.4)	0.169
Severe preeclampsia	2279 (1.1)	1943 (1.0)	336 (2.5)	0.114
Anemia	47080 (22.2)	43552 (22.0)	3528 (25.9)	0.092
Moderate or severe anemia	7583 (3.6)	6892 (3.5)	691 (5.1)	0.079
Liver-related diseases	8321 (3.9)	7605 (3.8)	716 (5.3)	0.068
Thyroid-related diseases	19323 (9.1)	17463 (8.8)	1860 (13.7)	0.154

*Liver-related diseases included intrahepatic cholestasis, hepatitis, liver dysfunction, liver damage etc., thyroid-related diseases included hyperthyroidism, hypothyroidism, thyroiditis, thyroid tumor, etc. ART, assisted reproductive technology.*

**TABLE 3:** Perinatal complications between naturally pregnancy and ART pregnancy.

Perinatal complications	All pregnancy (*n* = 211606)	Naturally pregnancy (*n* = 198002)	ART pregnancy (*n* = 13604)	Standardized difference
Hospitalization time (day)	5.6 ± 2.3	5.6 ± 2.3	6.2 ± 2.6	0.274
Preterm birth [n (%)]	16054 (7.6)	13274 (6.7)	2780 (20.4)	0.409
Premature rupture of membrane [n (%)]	58537 (27.7)	55583 (28.1)	2954 (21.7)	0.147
Amniotic fluid pollution [n (%)]				0.178
Clear (0°)	134056 (83.0)	124330 (82.6)	9726 (88.6)	
I°	8630 (5.3)	8164 (5.4)	466 (4.2)	
II°	8872 (5.5)	8484 (5.6)	388 (3.5)	
III°	9944 (6.2)	9546 (6.3)	398 (3.6)	
Polyhydramnios [n (%)]	5697 (2.7)	5236 (2.6)	461 (3.4)	0.044
Oligohydramnios [n (%)]	13656 (6.5)	12693 (6.4)	963 (7.1)	0.027
Cord entanglement [n (%)]	76217 (36.0)	71562 (36.1)	4655 (34.2)	0.040
Torsion of cord [n (%)]	6522 (3.1)	6073 (3.1)	449 (3.3)	0.013
Intrapartum fever [n (%)]	23815 (11.3)	22529 (11.4)	1286 (9.5)	0.063
Placenta previa [n (%)]	11483 (5.4)	10328 (5.2)	1155 (8.5)	0.130
Antepartum hemorrhage [n (%)]	557 (0.3)	513 (0.3)	44 (0.3)	0.012
Postpartum hemorrhage [n (%)]	22586 (10.7)	19979 (10.1)	2607 (19.2)	0.259
Cesarean section [n (%)]	89560 (42.7)	79317 (40.5)	10243 (75.5)	0.759

*ART, assisted reproductive technology.*

**TABLE 4:** Neonatal outcomes between naturally pregnancy and ART pregnancy.

Neonatal outcomes	All pregnancy (*n* = 211606)	Naturally pregnancy (*n* = 198002)	ART pregnancy (*n* = 13604)	Standardized difference
Gestational weeks	38.7 ± 1.9	38.8 ± 1.8	37.7 ± 2.4	0.519
**Gender [n (%)]**				
Boy	90392 (52.2)	83808 (52.2)	6584 (51.9)	0.098
Girl	82813 (47.8)	76713 (47.8)	6100 (48.1)	0.102
Birth weight (g)	3321.2 ± 507.9	3334.2 ± 495.2	3139.6 ± 632.2	0.343
Macrosomia [n (%)]	15301 (7.2)	14496 (7.3)	805 (5.9)	0.056
Twins or multiples [n (%)]	5201 (2.5)	2387 (1.2)	2814 (20.7)	0.657
Fetal distress [n (%)]	12956 (6.8)	12246 (6.9)	710 (5.5)	0.059
Stillbirth or abnormal development [n (%)]	2299 (1.1)	1845 (0.9)	454 (3.3)	0.167

*ART, assisted reproductive technology.*

Multivariable logistic regression analysis showed that the association between ART and pregnancy outcomes were significant (Table 5). Nearly all the pregnancy complication listed, including gestational diabetes [aOR (95%CI) = 1.39 (1.33–1.46)], preeclampsia [aOR (95%CI) = 1.26 (1.14–1.41)], moderate or severe anemia [aOR (95%CI) = 1.20 (1.08–1.32)], liver-related diseases [aOR (95%CI) = 1.14 (1.03–1.26)], and thyroid-related diseases [aOR (95%CI) = 1.29 (1.21–1.37)], were more likely to occur among women conceived by ART. In terms of perinatal complications, the risk of preterm birth [aOR (95%CI) = 1.61 (1.49–1.74)], placenta previa [aOR (95%CI) = 1.48 (1.37–1.60)], postpartum hemorrhage [aOR (95%CI) = 1.14 (1.08–1.21)], and cesarean section [aOR (95%CI) = 2.84 (2.70–2.99)] were significantly increased, while the risk of premature rupture of membrane [aOR (95%CI) = 0.66 (0.62–0.71)], amniotic fluid pollution [I°: aOR (95%CI) = 0.83 (0.74–0.92); II°: aOR (95%CI) = 0.66 (0.59–0.74); III°: aOR (95%CI) = 0.57 (0.51–0.64)], cord entanglement [aOR (95%CI) = 0.89 (0.85–0.93)], and intrapartum fever [aOR (95%CI) = 0.69 (0.64–0.74)] were significantly decreased in ART pregnancy as compared with naturally pregnancy. For neonatal outcomes, women conceived by ART were more likely to have twins or multiples [aOR (95%CI) = 24.20 (22.43–26.11)], and the risk of stillbirth or abnormal development [aOR (95%CI) = 2.76 (2.39–3.17)] was also significantly increased. Moreover, the risk of macrosomia [aOR (95%CI) = 0.88 (0.80–0.95)] and fetal distress [aOR (95%CI) = 0.66 (0.60–0.73)] were significantly decreased in ART pregnancy (Table 5).

**TABLE 5:** The association of ART with maternal and offspring health.

		Univariate		Multivariate	
Maternal and offspring health	Naturally pregnancy	ART pregnancy	*P*	ART pregnancy	*P*
**Pregnancy complications**					
Gestational diabetes (*n* = 39498)	1.0 (ref)	1.77 (1.70–1.84)	<0.001	1.39 (1.33–1.46)	<0.001
Preeclampsia (*n* = 5122)	1.0 (ref)	2.54 (2.35–2.75)	<0.001	1.26 (1.14–1.41)	<0.001
Severe preeclampsia (*n* = 2279)	1.0 (ref)	2.56 (2.27–2.87)	<0.001	1.12 (0.96–1.31)	0.146
Anemia (*n* = 47080)	1.0 (ref)	1.24 (1.19–1.29)	<0.001	1.01 (0.96–1.06)	0.678
Moderate or severe anemia (*n* = 7583)	1.0 (ref)	1.48 (1.37–1.61)	<0.001	1.20 (1.08–1.32)	<0.001
Liver-related diseases (*n* = 8321)	1.0 (ref)	1.39 (1.29–1.50)	<0.001	1.14 (1.03–1.26)	0.012
Thyroid-related diseases (*n* = 19323)	1.0 (ref)	1.64 (1.56–1.72)	<0.001	1.29 (1.21–1.37)	<0.001
**Perinatal complications**					
Preterm birth (*n* = 16054)	1.0 (ref)	3.58 (3.42–3.74)	<0.001	1.61 (1.49–1.74)	<0.001
Premature rupture of membrane (*n* = 58537)	1.0 (ref)	0.71 (0.68–0.74)	<0.001	0.66 (0.62–0.71)	<0.001
**Amniotic fluid pollution**					
I° (*n* = 8630)	1.0 (ref)	0.73 (0.66–0.80)	<0.001	0.83 (0.74–0.92)	0.001
II° (*n* = 8872)	1.0 (ref)	0.58 (0.53–0.65)	<0.001	0.66 (0.59–0.74)	<0.001
III° (*n* = 9944)	1.0 (ref)	0.53 (0.48–0.59)	<0.001	0.57 (0.51–0.64)	<0.001
Polyhydramnios (*n* = 5697)	1.0 (ref)	1.29 (1.17–1.42)	<0.001	1.00 (0.89–1.13)	0.992
Oligohydramnios (*n* = 13656)	1.0 (ref)	1.11 (1.04–1.19)	0.002	1.00 (0.92–1.08)	0.968
Cord entanglement (*n* = 76217)	1.0 (ref)	0.92 (0.89–0.95)	<0.001	0.89 (0.85–0.93)	<0.001
Torsion of cord (*n* = 6522)	1.0 (ref)	1.08 (0.98–1.19)	0.128	0.93 (0.83–1.05)	0.235
Intrapartum fever (*n* = 23815)	1.0 (ref)	0.81 (0.77–0.86)	<0.001	0.69 (0.64–0.74)	<0.001
Placenta previa (*n* = 11483)	1.0 (ref)	1.69 (1.58–1.80)	<0.001	1.48 (1.37–1.60)	<0.001
Antepartum hemorrhage (*n* = 557)	1.0 (ref)	1.25 (0.92–1.70)	0.157	0.82 (0.50–1.34)	0.421
Postpartum hemorrhage (*n* = 22586)	1.0 (ref)	2.11 (2.02–2.21)	<0.001	1.14 (1.08–1.21)	<0.001
Cesarean section (*n* = 79317)	1.0 (ref)	4.53 (4.35–4.72)	<0.001	2.84 (2.70–2.99)	<0.001
**Neonatal outcomes**					
Macrosomia (*n* = 15301)	1.0 (ref)	0.80 (0.74–0.86)	<0.001	0.88 (0.80–0.95)	0.002
Twins or multiples (*n* = 5201)	1.0 (ref)	21.37 (20.17–22.65)	<0.001	24.20 (22.43–26.11)	<0.001
Fetal distress (*n* = 12956)	1.0 (ref)	0.78 (0.72–0.84)	<0.001	0.66 (0.60–0.73)	<0.001
Stillbirth or abnormal development (*n* = 2299)	1.0 (ref)	3.67 (3.31–4.07)	<0.001	2.76 (2.39–3.17)	<0.001

*All values are ORs (95% CIs). Values were determined by using logistic regression. Adjusted values were adjusted for maternal age, intrapartum BMI, parity, birth plurality and abnormal pregnancy history. For odds of twins or multiples, adjusted values were adjusted for maternal age, intrapartum BMI, parity and abnormal pregnancy history.*

The association of ART with maternal and neonatal outcomes were also evaluated by stratifying on birth plurality and maternal age (Tables 6, 7). When restriction to singletons, the risks of adverse pregnancy outcomes as listed above were reduced. And the effects of ART on the risk of premature rupture of membrane, cord entanglement, intrapartum fever, cesarean section, and stillbirth or abnormal development (ART pregnancy vs. naturally pregnancy) were more pronounced among singleton pregnancies compared with that among pregnancies of twins or multiples, while the effect of ART on the risk of polyhydramnios was more prominent among pregnancies of twins or multiples (heterogeneity test: *P* < 0.05). When stratified by maternal age, we found the effects of ART on the risk of preterm birth, placenta previa, postpartum hemorrhage and cesarean section (ART pregnancy vs. naturally pregnancy) were more pronounced among women under 35 years compared with that among women over 30 years, while the effect of ART on the risk of polyhydramnios was more prominent among women over 35 years (heterogeneity test: *P* < 0.05).

**TABLE 6:** Stratified analysis on the association of ART with maternal and offspring health by birth plurality.

		ART pregnancy		
Maternal and offspring health	Naturally pregnancy	Singletons	Twins or multiples	*P*
**Pregnancy complications**				
Gestational diabetes	1.0 (ref)	1.40 (1.33–1.47)	1.34 (1.12–1.61)	0.648
Preeclampsia	1.0 (ref)	1.27 (1.12–1.43)	1.24 (0.99–1.56)	0.856
Severe preeclampsia	1.0 (ref)	1.11 (0.92–1.34)	1.10 (0.81–1.48)	0.960
Anemia	1.0 (ref)	1.00 (0.94–1.05)	1.15 (0.99–1.33)	0.082
Moderate or severe anemia	1.0 (ref)	1.17 (1.05–1.31)	1.35 (1.02–1.79)	0.353
Liver-related diseases	1.0 (ref)	1.17 (1.04–1.30)	1.06 (0.82–1.37)	0.489
Thyroid-related diseases	1.0 (ref)	1.30 (1.22–1.39)	1.21 (0.98–1.50)	0.528
**Perinatal complications**				
Preterm birth	1.0 (ref)	1.64 (1.49–1.80)	1.44 (1.24–1.66)	0.142
Premature rupture of membrane	1.0 (ref)	0.65 (0.61–0.70)	0.91 (0.71–1.16)	0.010
**Amniotic fluid pollution**				
I°	1.0 (ref)	0.83 (0.74–0.93)	0.81 (0.45–1.47)	0.937
II°	1.0 (ref)	0.67 (0.60–0.76)	0.40 (0.18–0.90)	0.214
III°	1.0 (ref)	0.58 (0.52–0.65)	0.29 (0.13–0.67)	0.101
Polyhydramnios	1.0 (ref)	1.09 (0.96–1.24)	0.72 (0.53–0.97)	0.013
Oligohydramnios	1.0 (ref)	1.02 (0.94–1.10)	0.96 (0.68–1.36)	0.738
Cord entanglement	1.0 (ref)	0.88 (0.84–0.92)	1.08 (0.93–1.26)	0.011
Torsion of cord	1.0 (ref)	0.96 (0.85–1.09)	0.68 (0.45–1.03)	0.118
Intrapartum fever	1.0 (ref)	0.68 (0.63–0.73)	1.09 (0.82–1.47)	0.002
Placenta previa	1.0 (ref)	1.46 (1.35–1.58)	1.96 (1.28–3.00)	0.183
Antepartum hemorrhage	1.0 (ref)	0.63 (0.35–1.15)	3.86 (0.73–20.25)	0.044
Postpartum hemorrhage	1.0 (ref)	1.17 (1.10–1.25)	1.04 (0.90–1.21)	0.152
Cesarean section	1.0 (ref)	2.92 (2.77–3.08)	1.23 (0.93–1.63)	<0.001
**Neonatal outcomes**				
Fetal distress	1.0 (ref)	0.69 (0.62–0.76)	0.55 (0.39–0.78)	0.219
Stillbirth or abnormal development	1.0 (ref)	3.48 (3.01–4.03)	0.82 (0.60–1.14)	<0.001

*All values are ORs (95% CIs). Values were determined by using logistic regression. Adjusted values were adjusted for maternal age, intrapartum BMI, parity and abnormal pregnancy history. The P-values were calculated for heterogeneity test.*

**TABLE 7:** Stratified analysis on the association of ART with maternal and offspring health by maternal age.

		ART pregnancy		
Maternal and offspring health	Naturally pregnancy	Maternal age < 35 y	Maternal age ≥ 35 y	*P*
**Pregnancy complications**				
Gestational diabetes	1.0 (ref)	1.59 (1.50 – 1.68)	1.45 (1.31 – 1.60)	0.116
Preeclampsia	1.0 (ref)	1.34 (1.19 – 1.52)	1.19 (0.94 – 1.50)	0.378
Severe preeclampsia	1.0 (ref)	1.15 (0.96 – 1.37)	1.14 (0.82 – 1.58)	0.963
Anemia	1.0 (ref)	1.02 (0.97 – 1.08)	1.02 (0.91 – 1.15)	1.000
Moderate or severe anemia	1.0 (ref)	1.27 (1.13 – 1.41)	1.24 (0.97 – 1.58)	0.861
Liver-related diseases	1.0 (ref)	1.15 (1.02 – 1.29)	1.18 (0.95 – 1.47)	0.839
Thyroid-related diseases	1.0 (ref)	1.32 (1.23 – 1.42)	1.39 (1.21 – 1.60)	0.519
**Perinatal complications**				
Preterm birth	1.0 (ref)	1.56 (1.43 – 1.70)	1.26 (1.06 – 1.51)	0.034
Premature rupture of membrane	1.0 (ref)	0.70 (0.65 – 0.75)	0.67 (0.57 – 0.80)	0.641
**Amniotic fluid pollution**				
I°	1.0 (ref)	0.81 (0.72 – 0.91)	0.96 (0.74 – 1.24)	0.240
II°	1.0 (ref)	0.68 (0.60 – 0.78)	0.71 (0.53 – 0.94)	0.788
III°	1.0 (ref)	0.62 (0.54 – 0.70)	0.53 (0.38 – 0.74)	0.390
Polyhydramnios	1.0 (ref)	0.94 (0.82 – 1.07)	1.40 (1.11 – 1.77)	0.004
Oligohydramnios	1.0 (ref)	1.03 (0.94 – 1.13)	0.93 (0.76 – 1.12)	0.351
Cord entanglement	1.0 (ref)	0.91 (0.87 – 0.96)	0.86 (0.78 – 0.96)	0.335
Torsion of cord	1.0 (ref)	0.98 (0.86 – 1.11)	1.04 (0.78 – 1.37)	0.706
Intrapartum fever	1.0 (ref)	0.74 (0.68 – 0.80)	0.78 (0.66 – 0.93)	0.587
Placenta previa	1.0 (ref)	1.77 (1.61 – 1.94)	1.24 (1.06 – 1.44)	<0.001
Antepartum hemorrhage	1.0 (ref)	0.89 (0.51 – 1.56)	0.80 (0.29 – 2.25)	0.858
Postpartum hemorrhage	1.0 (ref)	1.27 (1.19 – 1.35)	1.07 (0.94 – 1.22)	0.020
Cesarean section	1.0 (ref)	3.17 (3.00 – 3.35)	2.39 (2.08 – 2.76)	<0.001
**Neonatal outcomes**				
Fetal distress	1.0 (ref)	0.75 (0.68 – 0.83)	0.58 (0.45 – 0.75)	0.066
Stillbirth or abnormal development	1.0 (ref)	2.48 (2.13 – 2.88)	2.25 (1.52 – 3.32)	0.649

*All values are ORs (95% CIs). Values were determined by using logistic regression. Adjusted values were adjusted for intrapartum BMI, parity, birth plurality and abnormal pregnancy history. The P-values were calculated for heterogeneity test.*

## Discussion

This retrospective, hospital-based cohort study including 13,604 ART pregnancies and 198,002 naturally pregnancies was conducted in Nanjing, China from 2011 to 2020. The study showed the widespread application of ART in China, with the proportion of ART pregnancies increasing year by year in the past decade, and confirmed the increased risks of several adverse pregnancy outcomes in ART pregnancies. We found a 24.2-fold increase in the incidence of multiple births in ART pregnancies compared to naturally pregnancies, then stratified the analysis by birth plurality, suggesting multiple births are indeed an important factor leading to adverse pregnancy outcomes.

In the present study, the increased risks were found in ART pregnancy compared with naturally pregnancy: gestational diabetes (1.39-fold), preeclampsia (1.26-fold), moderate or severe anemia (1.20-fold), liver-related diseases (1.14-fold), thyroid-related diseases (1.29-fold), preterm birth (1.61-fold), placenta previa (1.48-fold), postpartum hemorrhage (1.14-fold), cesarean section (2.84-fold), and stillbirth or abnormal development (2.76-fold), which were largely consistent with the findings of previous studies (10, 28–32). Although these risks were reduced when restriction to singletons, significant differences remained. Some studies have suggested that infertility is one of the risk factors for adverse pregnancy outcomes (33). However, infertility factors cannot fully explain the associations. For infertile women, women conceived with ART had an increased risks of adverse pregnancy outcomes compared with women conceived with non-ART (34). Therefore, some researchers believe that the increased risks of adverse outcomes after ART conception are mainly related to ART manipulation factors (6), which is due to the addition of many non-physiological operations by ART. For example, the type of ovulation induction drugs used in the early stage, the composition of the culture medium, the storage time in the culture medium, the freezing and dissolution process of the embryo, polyspermic fertilization, and the hormone level at the time of implantation, all play an important role in the occurrence of adverse pregnancy outcomes (35). Other studies have pointed out that different methods of ART may lead to different types of adverse pregnancy outcomes (36). At the same time, the longer and more times of ART treatment, the greater the harm to women and their offspring (36). In addition, ART pregnancies may be more closely monitored than naturally pregnancies, which partly explains the higher incidence of adverse pregnancy outcomes in ART pregnancies (37). However, current studies and evidence cannot fully elucidate the mechanism by which ART increases the risk of adverse pregnancy outcomes, and the specific mechanism needs further research.

The main advantage of this study was the large sample size of pregnancy, which allowed us to conduct further subgroup analysis with enough power. And the data obtained from HIS database by using NLP technique is of high quality. However, there are some limitations in this study. First, the population we studied was limited to one city in eastern China (Nanjing). Therefore, we should be cautious in generalizing our findings to other regions. Second, we did not collect information on the ART form. The more intricate and invasive the ART form used, the more likely it was to cause adverse pregnancy outcomes. And the records of pre-pregnancy BMI, baseline endocrine level, causes of infertility, ovarian stimulation protocols, and quality of transferred embryos lacked in our database, were not included in this study. Third, the retrospective design of this study could not assess a causal relationship between ART and adverse pregnancy outcomes. These limitations should be considered in future studies.

## Conclusion and Prospect

Women conceived by ART were at increased risks of several adverse pregnancy outcomes compared with women conceived naturally. Multiple pregnancies due to multiple embryos transferred could partly explain the increased risks. The transfer of single embryo of high quality should be promoted. However, ART singleton pregnancy still showed higher risks of several adverse pregnancy outcomes compared with naturally pregnancy, suggesting ART itself is also significantly correlated with pathological pregnancy. Therefore, policies related to ART indications should be strictly formulated to reverse the high rate of ART pregnancy. Given our findings, prenatal and intrapartum monitoring should be strengthened, and neonatal outcomes should be closely observed for ART pregnancy. And more research should be conducted to further clarify whether the increased risk of adverse pregnancy outcomes is due to ART itself, multiple births, or potential infertility.

## Data Availability Statement

The original contributions presented in the study are included in the article/supplementary material, further inquiries can be directed to the corresponding authors.

## Ethics Statement

The studies involving human participants were reviewed and approved by the institutional review board of Women’s Hospital of Nanjing Medical University (2020KY-011). Written informed consent for participation was not required for this study in accordance with the national legislation and the institutional requirements.

## Author Contributions

JW initiated, conceived, and supervised the study. WT and LH did data collection and performed the data analysis. All authors approved the final format of the submitted manuscript.

## Conflict of Interest

The authors declare that the research was conducted in the absence of any commercial or financial relationships that could be construed as a potential conflict of interest.

## Publisher’s Note

All claims expressed in this article are solely those of the authors and do not necessarily represent those of their affiliated organizations, or those of the publisher, the editors and the reviewers. Any product that may be evaluated in this article, or claim that may be made by its manufacturer, is not guaranteed or endorsed by the publisher.

## Abbreviations

ART: assisted reproductive technology
ICSI: intracytoplasmic sperm injection
IVF: *in vitro* fertilization
OI: ovulation induction
GIFT: gamete intra-fallopian transfer
AI: artificial insemination
HIS: hospital information system
NLP: natural language processing
BMI: body mass index
OR: odds ratio
CI: confidence intervals.
